# Historical Spatial Distribution of Zoonotic Diseases in Domestic, Synanthropic, and Wild Animals in the Mexican Territory of the Yucatan Peninsula

**DOI:** 10.1155/2021/8699455

**Published:** 2021-08-07

**Authors:** Paulina Haro, Enrique Trasviña-Muñoz, Irving May-Concha, Gilberto López-Valencia, Francisco Monge-Navarro, Carloman Herrera-Ramírez, Julio A. Mercado-Rodríguez, Hernán Villanueva-Alonzo, Etienne Waleckx

**Affiliations:** ^1^Instituto de Investigaciones en Ciencias Veterinarias, Universidad Autónoma de Baja California, Mexicali, Mexicali, Mexico; ^2^Laboratorio de Parasitología, Centro de Investigaciones Regionales Dr. Hideyo Noguchi, Universidad Autónoma de Yucatán, Mérida, Mérida, Mexico; ^3^Laboratorio de Biología Celular, Centro de Investigaciones Regionales Dr. Hideyo Noguchi, Universidad Autónoma de Yucatán, Mérida, Mérida, Mexico; ^4^Institut de Recherche pour le Développement, UMR INTERTRYP IRD, CIRAD, Université de Montpellier, Montpellier, France

## Abstract

The Mexican territory of the Yucatan Peninsula has a tropical climate and harbors a wide variety of domestic, synanthropic, and wild animals, as well as disease vectors. To determine the distribution of recorded zoonotic diseases in the Yucatan Peninsula, scientific publications referring to these diseases in animals and containing geographic coordinates of disease occurrence, were studied. The epidemiological bulletins of the national government were also consulted to obtain information on zoonotic diseases reported in humans in the territory. The territory harbors a wide variety of tropical zoonotic pathogens, including *Trypanosoma cruzi*, *Leptospira interrogans, Toxoplasma gondii*, *Leishmania mexicana*, *Dirofilaria immitis*, and *Rickettsia felis.* A variety of domestic, synanthropic, and wild animals act as hosts or reservoirs in the transmission cycle of the zoonotic diseases in the Yucatan Peninsula, and some spillover into human populations has also been recorded. There are still zoonotic diseases that have rarely or never been reported in humans, but it is not clear whether this is because these diseases in humans are not common, there is a lack of viable transmission cycle or there is a lack of appropriate diagnosis. It is necessary to continue monitoring vectors, animal hosts, and humans to identify risk factors for zoonotic diseases in the Yucatan Peninsula.

## 1. Introduction

Zoonotic infectious diseases (or zoonoses) are communicable diseases that are transmitted from animals to humans [1]. They can pose serious risks to both animal and human health and may have far-reaching economic impacts. Zoonoses can be food-borne, waterborne, vector-borne, transmitted through direct contact with animals (or indirectly by fomites), or transmitted by environmental contamination [2]. These diseases have been important concerns for humans since the beginning of the domestication of animals 10,000 years ago [3] and remain a major public health issue worldwide. Approximately 75% of emerging infectious diseases are zoonoses [4], and just 13 zoonoses (top-ranked as priority by the WHO) are responsible for an estimated 2.2 million human deaths and 2.4 billion cases of illness in humans per year around the world [5]. It has been estimated that more than six out of every ten known infectious diseases in humans—and three out of every four new or emerging infections—are spread from animals [6].

The Mexican territory of the Yucatan Peninsula, which includes the states of Campeche, Quintana Roo, and Yucatan, has been occupied by human populations for at least the last 12,000–13,000 years [7, 8]. It is a biogeographical area with a subhumid, warm tropical climate and lies between the Gulf of Mexico to the north and northwest, the Caribbean Sea to the southeast, and Belize and Guatemala to the south. The human population of the Yucatan Peninsula grew from 1.7 million in 1980 to 4.5 million in 2015 [9], and it was estimated to have reached over five million by 2020 [10]. Between 75% and 88% of the population live in urban environments [11], and there are strong social inequalities, with a poverty rate estimated between 28.8% and 43.8% [12, 13]. Together, these factors create an ideal environment for the occurrence of emerging and reemerging zoonoses [14], and Chagas disease, leishmaniasis, and rickettsial infections remain important health issues in this area [15]. This study was conducted to synthesize cases of zoonotic diseases reported between 1995 and 2019 in the Mexican territory of the Yucatan Peninsula and highlight their geographical distributions.

## 2. Materials and Methods

### 2.1. Criteria for Inclusion of Studies on Animals in the Database

Potential data sources were identified from ISI's Web of Science (v5.13.1), which incorporates many relevant databases, including the SciELO Citation Index from 1997 onwards (providing access to leading journals from Latin America, Portugal, Spain, and South Africa) and the Web of Science's Core Collection from 1980 onwards (https://webofknowledge.com/). Studies were also selected from the NCBI PubMed database (https://www.ncbi.nlm.nih.gov/pubmed/) and Scopus (https://www.scopus.com).

We restricted our search to the period from 1995 to June of 2019 and looked for cases of zoonotic diseases diagnosed in domestic and wild animals in the Mexican territory of the Yucatan Peninsula that included geographical coordinates of the sampling area. We first performed a general search using the major query term, “Zoonoses” and then filtered the results using the sentences “Animal zoonoses in the Yucatan Peninsula Mexico” and “Zoonosis en los animales de la Peninsula de Yucatan Mexico.”

### 2.2. Inclusion Criteria

The articles were screened for any indication that the study contained data related to evidence of zoonosis infection in animal populations and geographical coordinates of sampling.

The information collected was (1) publication data (bibliographic information); (2) sampling dates; (3) Yucatan Peninsula state (Campeche, Quintana Roo, and Yucatan); (4) locality of collection (name of the city or village); (5) geographical coordinates of sampling; (6) sample size; (7) studied population (domestic or wild); (8) name of animal studied; (9) environment (domestic or wild animal); (10) name of zoonotic infectious diseases detected; (11) number of cases diagnosed; (12) prevalence.

### 2.3. Criteria for Inclusion of Studies on Humans in the Database

Only reports from the government sector (the Historical Epidemiological Bulletin, produced by the Ministry of Health) were included. The reports list cases of diseases associated with transmission between humans and animals from 2008 to 2019. Data from week 52 of each considered year, or the cumulative number of cases, were included [16].

## 3. Results

**Figure 1:**
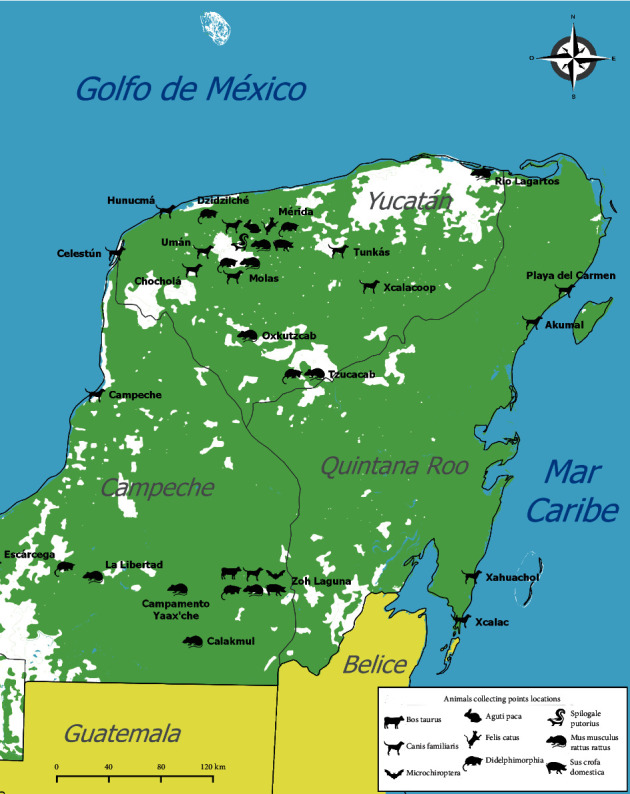
Yucatan Peninsula with georeferenced locations of samples from domestic, synanthropic, and wild animals carrying a zoonotic disease.

A total of 35 scientific publications on zoonotic diseases in animals in the Yucatan Peninsula containing collecting point coordinates of zoonotic diseases were found. Twenty-two locations were identified, including 12 localities of the state of Yucatan, 6 localities of the state of Campeche, and 4 localities of the state of Quintana Roo (Figure 1).

**Figure 2:**
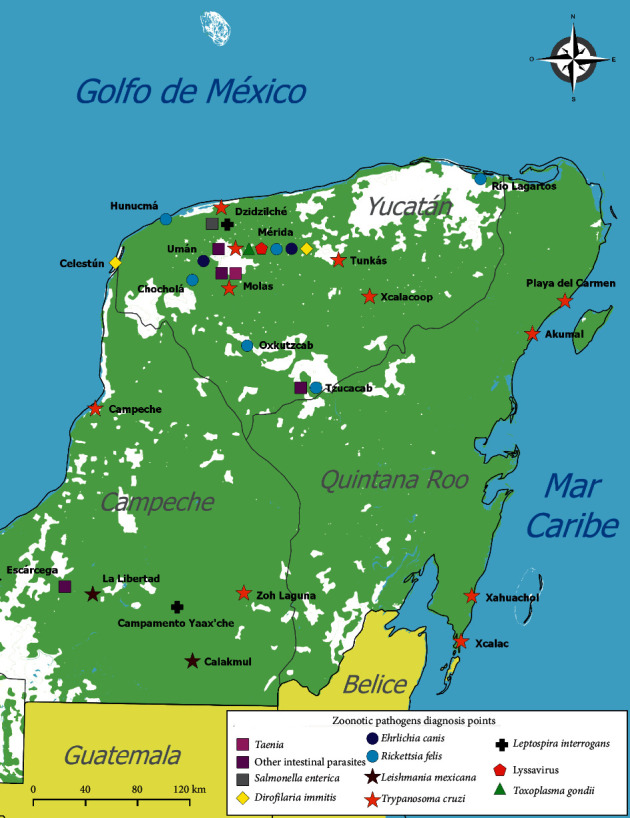
Yucatan Peninsula with georeferenced locations for zoonotic pathogens diagnosed in animals.

The main zoonotic pathogens detected were *Trypanosoma cruzi*, *Toxoplasma gondii*, *Leishmania mexicana*, *Dirofilaria immitis*, *Rickettsia felis*, *Cysticercus fasciolaris, Ehrlichia canis*, lyssaviruses, *Leptospira interrogans, Salmonella enterica*, *Ancylostoma caninum*, *Trichuris vulpis*, *Toxocara canis*, *Dipylidium caninum*, *Cruzia tentaculata*, *Turgida turgida,* and other zoonotic intestinal parasites (Figure 2).

The following diagnostic techniques were employed singly or in combination: enzyme-linked immunosorbent assay, indirect immunofluorescence assay, indirect haemagglutination, western blot, polymerase chain reaction, histopathology, blood smear, and microscopy agglutination.

Domestic or synanthropic animals associated with zoonotic diseases in the Yucatan Peninsula were from the following taxa: order Carnivora, Canidae (*Canis familiaris*), Felidae (*Felis catus*); order Rodentia (*Rattus rattus* and *Mus musculus*); order Artiodactyla, Bovidae (*Bos taurus*), and Suidae (*Sus domesticus*); order Didelmorphia, Marsupialia (*Didelphis virginiana and Didelphis marsupialis*). Taxa of wild animals associated with zoonotic diseases in the Yucatan Peninsula were as follows: order Carnivora, Mephitidae (*Spilogale putorius*); order Rodentia (*Peromyscus yucatanicus*, *Sigmodon hispidus*, *Ototylomis phyllotis*, *Heteromys gaumeri*, *Heteromys desmarestianus*, *Oryzomys melanotis*, *Oryzomys couesi*, *Oligoryzomys* spp., and *Reithrodontomys gracilis*); order Cuniculidae (*Agouti paca*); order Didelphimorphia, Marsupialia (*Marmosa mexicana and Philander opossum*); order Chiroptera (*Artibeus jamaicensis, Artibeus lituratus, Dermanura phaotis, Sturnira lilium,* and *Sturnira Ludovici*) (Figure 1).

*Canis familiaris*, *Mus musculus*, *Didelphis virginiana*, and *Felis catus* were the four species of domestic or synanthropic animals most commonly associated with zoonotic diseases in the Yucatan Peninsula (Table 1). Regarding zoonotic pathogens most frequently diagnosed, in dogs, the most common diseases were *Trypanosoma cruzi (T. cruzi)* and *Dirofilaria immitis (D. immitis)*. In rodents, the most frequent diseases were *Leishmania mexicana (L. mexicana)*, *Leptospira spp.*, and *T. cruzi*. Opossums were related to *T. cruzi* and *Leptospira spp.* Finally cats were associated with *Toxoplasma gondii (T. gondii)* and *T. cruzi* (Table 2).

In the Yucatan Peninsula, most of the studies involved murids. Wild environment collected rodents were associated with genus *Leptospira*, *Leishmania*, *Rickettsia*, *Trypanosoma*, *Taenia*, and domestic environment collected rodents with *Rickettsia* and *Trypanosoma*.

Genus *Leptospira* was found in wild rodents with a prevalence of 15% (9/60); the predominant serotypes were *icterohaemorrhagiae*, *wolffi*, and *bratislava* [17]. Espinosa-Martinez et al. reported wild rodent species *Heteromys gaumeri* and *Ototylomys phyllotis* as new carriers of *Leptospira interrogans* [18]. Antibodies against *L. mexicana* were detected in the following wild rodent species: *O. melanotis, O. phyllotis, P. yucatanicus*, and *Sigmodon hispidus* [19, 20]. *L. mexicana* was reported by Van Wynsberghe et al. [21, 22], in association with *Heteromys gaumeri, Heteromys desmarestianus, O. phyllotis, P. yucatanicus, Sigmodon hispidus, O. melanotis*, and *Reithrodontomys gracilis*. It is notable that 50–56% of the rodents presented the infection asymptomatically and thereby acted solely as reservoirs. It has been hypothesized that the multiplication of parasites in *P. yucatanicus* might be triggered by high temperature. It has also been reported that high humidity and low temperatures promote populations of sand flies, which act as vectors for this pathogen in rodents. Andrade-Narvaez et al. suggested that *Lutzomyia olmeca olmeca* was one of the most likely vectors for rodents [23].

One of the main studies of *Rickettsia felis (R. felis)* in small mammals in wild and domestic areas was conducted by Panti-May et al., who found the wild rodent species *O. phyllotis, H. gaumeri*, *S. hispidus*, *P. yucatanicus*, and *Oligorizomys sp.* were infected, though *Mus musculus* was the only synanthropic species carrying the infection [24]. The vectors of *R. felis* were the fleas *Polygenis odiosus* (collected from *Ototylomys phyllotis*) and *Ctenocephalides felis* (collected from *Peromyscus yucatanicus*), while *Rickettsia typhi* was detected in blood samples obtained from *Rattus rattus* [25].

*T. cruzi* was detected in the domestic species *R. rattus* and *M. musculus* and the wild species *P. yucatanicus, Peromyscus leucopus, Dasyprocta punctate*, and *Urocyon cinereoargenteus*; *R. rattus* presented the highest seroprevalence in nondomestic collection environment [26]. In a 2015 study, Lopez-Cancino et al. found a 50% (4/8) prevalence of *T. cruzi* in rodents [27].

*Taenia, Cysticercus fasciolaris (C. fasciolaris)*, was found with 7.5% (31/411) prevalence in domestic collected rodents. Liver cysts of *C. fasciolaris* were identified in *M. musculus* and *R. rattus*, and adults male mice were 4.33 and 3.46 (OR values) times more likely to be infected [28]. Mice are only intermediate hosts for *C. fasciolaris*; cats are the main definitive host, so the full extent of *C. fasciolaris* epidemiology is still unknown [29].

Specific antibodies against *T. cruzi* were found in fattening pigs from Yucatan, Mexico; between 273 sampled pigs, 5.4% (*n* = 15) were found positive [30]. Another study in pigs detected high seropositivity of genus *Leptospira*, with a prevalence of 25% (88/353); *bratislava, icterohaemorrhagiae*, and *panama* were the predominant serotypes [17].

Studies carried in dogs confirm this species to be one of the main carriers of *T. cruzi*. Lopez-Cespedes et al. reported a seropositivity of 14.76% (93/630) [31], and Zavala-Velázquez et al. reported 15.84% (29/183) in a study in which dogs had the highest seroprevalence among domestic animals [26]. Jiménez-Coello et al. detected the infection and reported no statistical difference between *T. cruzi* seroprevalence in stray dogs from an urban area (9.8%; 10/102) and rural dogs (17.3%; 42/243) [32]. In another study comparing the different population of dogs, an overall seroprevalence of 7.6% (20/262) was found, and there was a difference between stray dogs and dogs with owners, with the former having a higher seroprevalence (9.5%; 14/148 vs 5.3%; 6/114) [33]. Cruz-Chan et al. reported the presence of a possible coinfection, having detected antibodies of *T. cruzi* and *D. immitis* helminths, and these potentially coinfected animals had lower antibody values (IgM) [34]. Jiménez-Coello et al. detected a seroprevalence in *Canis familiaris* of 12.2% (45/370), and animals older than two years had a greater risk (*P* > 0.06) of becoming infected with *T. cruzi* than younger animals [35].

*Leptospira genus* in dogs was found with a prevalence of 19% (36/192), and the predominant serotypes were *grippotyphosa* and *pomona* [17]. The prevalence of *D. immitis* was 59.8% (167/279), and the age of individuals (>2 years) was a risk factor for infection (OR = 2.49) [36].

The seroprevalence of *Ehrlichia canis (E. canis)* in dogs screened in a 2009 study was 8.7% (27/309) [37], and in a recent study of stray dogs and house dogs using PCR as the diagnostic tool, the overall prevalence was 36% (18/50; only stray dogs were infected). The tick *Rhipicephalus sanguineus* was the vector implicated, and male ticks had a higher infection rate than females [38]. *Rickettsia akari* was found in a dog by sequencing, but although the mite *Liponyssoides sanguineus* is the only known vector associated with this pathogen, the clinical history only indicated contact with ticks [39]. This finding suggests that ticks could also serve as vectors for *R. akari*, but this could not be demonstrated in this study.

A study on intestinal parasites of 130 dogs in the Yucatan Peninsula found that 104 (80%) were positive for the presence of the parasites. *Ancylostoma caninum (A. caninum)* was the most prevalent species (73.8%; 96/130), followed by *Trichuris vulpis (T. vulpis)* (25.4%; 33/130), *Toxocara canis (T. canis)* (6.2%; 8/130), and *Dipylidium caninum (D. caninum)* (2.3%; 3/130). The majority of the dogs were infected by only one species of parasite (70/130, 53.8%); mixed infections caused by two and three zoonotic parasites were found in 21.3% (30/130) and 3.1% (4/130), respectively. *A. caninum* showed the highest egg output (42.3% of dogs had ≥ 500 eggs per gram). Dogs <2 years old were 5.30 (OR) times more likely to be infected with zoonotic intestinal parasites than dogs > 5 years old, and those with poor body conditions were 6.69 (OR) times more likely to be infected with zoonotic intestinal parasites than those with good body conditions [40]. Viral zoonotic disease in dogs was detected in the period from 1999 to 2002, including four cases of rabies from Yucatan, though the canine rabies detected did not appear to have any phylogenetic connections with other cases from the country [41]. One white-tailed deer (*Odocoileus virginianus*), two agoutis (*Agouti paca*) maintained in captivity, and one spotted skunk (*Spilogale putorius*) were found to be carrying the rabies virus [41].

Studies in cats in the peninsula indicate that these hosts are infected with *T. cruzi*; 34% (75/220) prevalence was detected using PCR, and older cats were more likely to carry the infection than younger cats [42]. *T. gondii* is one of the main zoonotic infections in cats worldwide, and in Yucatan, 79% (202/220) of in-house cats were found to be positive in a study using molecular techniques [43].

In the Peninsula, *T. cruzi* has been identified in opossums (*Didelphis marsupialis*), with a seroprevalence of 11.81% (13/110) [26]. The first report of *T. cruzi* in *Didelphis virginiana* using microscopy to detect the infection indicated a prevalence of 53.9% (55/102), while 16.2% (32/197) of the triatomine vectors were infected; the prevalence of infection in opossums is highest during the rainy season [44]. Another study reported an infection prevalence of 55% (21/38) in opossums and found a higher prevalence in adults than juveniles [45]. Other marsupials found infected with *T. cruzi* were *Marmosa mexicana* and *Philander opossum* [27]. Another important infection in opossums was *Leptospira*, detected with a seropositive rate of 5% (4/80), and the main serotypes were *pomona* and *wolffi* [17]. Rickettsial diseases were also detected in *Didelphis virginiana; R. felis* was found using PCR in 57% of animals tested (4/7) [24].

A study of opossums found 29.4% (5/17) infected with one of four *Salmonella enterica enterica* serotypes and one *Salmonella enterica* subsp. *arizonae*; this was the first report of *Salmonella enterica* in Yucatan [46]. Intestinal parasitic infections detected in *Didelphis virginiana* included *Cruzia tentaculata* and *Turgida turgida* [47].

Another study in the peninsula found bats to be one of the main carriers of *T. cruzi*; species infected were as follows: *Artibeus jamaicensis, Artibeus lituratus, Dermanura phaotis, Sturnira lilium,* and *Sturnira ludovici* [27].

### 3.1. Humans

Reports of zoonoses in humans from 2008 to 2019 were compiled from the database of the Mexican government sector [16] and covered the following diseases: leishmaniasis, trypanosomiasis, leptospirosis, Rocky Mountain spotted fever, other rickettsial diseases, and undefined intestinal diseases (Table 3). Cutaneous leishmaniasis was found to be one of the more commonly reported zoonoses in humans in the peninsula (1955 cases), but no cases of visceral leishmaniasis were reported. American trypanosomiasis was the second most frequent zoonotic disease, with a total of 798 cases, and 171 cases of rickettsial diseases were reported, with peaks of cases in the years 2015 and 2019. Notably, the government reports classify rickettsial diseases into two groups—Rocky Mountain fever and other rickettsial diseases—but do not specify what is contained in the latter category. There were 104 cases of leptospirosis and 75 cases of toxoplasmosis (records of which began in 2014). More than two million cases of undefined intestinal diseases were recorded (peaking in 2017), but it is not clear which diseases are involved or what proportion of cases may be related to zoonoses [16].

## 4. Discussion

To understand the occurrence of animal and human shared diseases best known as zoonoses, new concepts as “One Health” and “EcoHealth” have been developed. They aim to approach the link between environment, pathogens, humans, animals, and vectors involved in the occurrence of zoonotic diseases. Most studies on zoonotic diseases focus on the level of local populations and communities, and it is not common to find studies of larger scales, such as the landscape and regions that play an important role in infectious disease dynamics [48].

The Yucatan Peninsula has a tropical climate and harbors a wide variety of domestic, synanthropic, and endemic wild animals, as well as vectors such as ticks, mosquitoes, and bugs. These conditions are ideal for the cyclic transmission of a wide variety of tropical zoonotic diseases.

The present study is important because it synthesizes historical and geographic data on emerging and reemerging zoonoses in the Yucatan Peninsula and should improve mechanisms for the efficient circulation of information about these diseases between the different actors involved (society, scientific community, and public and private institutions), thereby helping to achieve effective prevention and control strategies that can become public health policies.

The information found in the present study demonstrates that the peninsula is home to a wide range of zoonotic protozoa, bacteria, viruses, and intestinal parasites, including *T. cruzi*, *T. gondii*, *L. mexicana*, *D. immitis*, *R. felis*, *C. fasciolaris*, *E. canis*, lyssaviruses, and *L. interrogans*. Although the information collected is limited to the Yucatan Peninsula, the perspectives presented in this study apply to different scales and geographic regions.

Among animals, evidence of the infection caused by *T. cruzi* was the most represented in the peninsula. This protozoan causes Chagas disease in humans, and the World Health Organization (2020) estimates that there are between 6 and 7 million human cases globally, with the majority in Latin American [49]. The main vector associated with the transmission of this disease in Yucatan is *Triatoma dimidiata* (*T. dimidiata*), and rodents, dogs, and opossums are the main hosts [50]. Bats are also known hosts and play an important role in the transmission of numerous parasites throughout the world [51]. The environmental conditions in the region, coupled with the presence of vectors and the diversity of mammalian hosts, make the Yucatan Peninsula suitable for the development of this parasite's life-cycle, as well as its onward transmission to humans and domestic animals. It is notable that, in the Yucatan Peninsula, it has been reported that there is no difference in prevalence between stray and house dogs, which may be due to the intrusive behavior of *T. dimidiata* in this region [52, 53], where an effective strategy for avoiding the disease is to prevent the vector's access to homes, especially via the use of windows screens [54, 55].

The prevalence of leptospirosis in animals implies a problem not only at an epidemiological level but also at an economic one [56]. Since it was observed in pigs, it could cause the death of these valuable animals. The main hosts reported were rodents, dogs, pigs, and opossums, all of which allow the continuation of the biological cycle of *Leptospira* in the region [57]. Although there were few human cases of leptospirosis reported, it is still necessary to identify the main risk factors that lead to transmission. Vado-Solis et al. reported that the main risk factors for humans in Yucatan were having rodents in the house or surroundings and being exposed to natural water deposits (*aguadas* and *cenotes*) that could be contaminated with animal urine [17].

Cutaneous leishmaniasis is transmitted by *mosca chiclera* (phlebotomine sand flies) and is associated with certain work activities, such as collecting the resin of the chicozapote tree (*Achras zapota* L.), the main ingredient in chewing gum, hence the name of “Chiclero's ulcer.” In the Yucatan Peninsula, the causative agent of the cutaneous form has been identified mainly as *Leishmania* (*Leishmania*) *mexicana* (Biagi), although, on a few occasions, the parasite *Leishmania* (*Viannia*) *braziliensis* (*Viannia*) has also been reported [58]. According to officially reported cases in the region, cutaneous leishmaniasis is the first most commonly reported zoonotic disease in humans in the Yucatan Peninsula (Table 3). Rodents were also the only mammals considered as carriers of *L. mexicana*, but we found no studies in other animal species, so it cannot be established whether other species serve as reservoirs.

The government reports categorize rickettsial diseases as either Rocky Mountain fever or other rickettsial diseases, and the latter designation prevented us from being able to identify the species and, therefore, discern the identity of vectors and reservoirs that participate in the biological cycle of these infectious agents.

Although *T. gondii* cases in humans were not numerous, the high prevalence of infection in cats revealed in this study should be a matter of public health concern, given the extent of direct contact between these reservoirs and the human population, and we recommend that the prevalence in rodents be measured.

The relatively low number of human cases of toxoplasmosis and other diseases discussed in this study might be partly explained by the fact that many zoonoses have symptoms that are easily confused with those of other febrile infections in the region [17].

Most of the studies on animals presented here measure prevalence via the detection of antibodies, and we recommend that further prevalence studies be conducted using more direct techniques, to more accurately identify reservoirs of neglected tropical diseases (NTDs). In addition, more detailed studies in humans should be considered in areas where diseases with potential zoonotic transmission have been identified in animals.

Although it was not the aim of the present study to establish a real estimation of the number of human cases in the region for each zoonotic disease, we must mention that the human cases officially informed on the Historical Epidemiological Bulletin are probably underestimated. For instance, concerning *T. cruzi* infection, a noticeable difference exists between the national official prevalence (0.65%) and the estimated prevalence obtained through a meta-analysis of recent research studies (3.38%) [59].

To establish effective diagnosis and control strategies, it is essential to understand the spatial and temporal distributions of vectors and reservoir hosts and elucidate their roles in transmission. These strategies should be collaborative efforts, spanning a set of diverse disciplines associated with health problems, and should include the beneficiaries as well as the researchers [60]. Finally, the emergence of severe acute respiratory syndrome coronavirus 2 (SARS-CoV-2), the causative agent of COVID-19, could represent a problem for future monitoring and study of NTDs, since it will divert much needed financial and human resources. It is even possible that some of the progress already achieved in NTD control and elimination efforts might be reversed [61, 62].

## 5. Conclusions

The territory of the Yucatan Peninsula presents a favorable environment for the survival of vectors and hosts that are essential for the transmission cycles of a wide range of zoonotic pathogens, including *T. cruzi*, *L. interrogans, T. gondii*, *L. mexicana*, *D. immitis*, and *R. felis*, all of which have been detected in a diversity of domestic, synanthropic, and wild animals, as well as humans. In animals, infection with *T. cruzi* was the most common zoonotic pathogen found. Cutaneous leishmaniasis was the most commonly reported zoonotic disease in humans. It should be emphasized there are zoonotic diseases for which few or no cases were reported in humans, raising the question of whether these diseases are not common in humans, the disease cycle cannot be accomplished, or there is simply a lack of appropriate diagnosis. However, it is necessary to conduct studies considering large scale vision of the diseases as metacommunity of mathematical models to study the interaction between pathogens, vectors, animal reservoirs, humans, no-host, and other concepts as environmental disruptors to gain a better understanding of zoonoses occurrence in the Yucatan Peninsula.

## Figures and Tables

**Table 1:** Yucatan Peninsula domestic, synanthropic, and wild animals and the zoonotic pathogens detected using direct and indirect diagnostic methods.

Order	Species	Environment of capture	Bacterial diseases	Parasitic diseases	Viral diseases
Rodentia	*Mus musculus and Rattus rattus*	Domestic/synanthropic	*Rickettsia felis*	*Trypanosoma cruzi*	
	Different species	Wild	*Leptospira interrogans, Rickettsia felis*	*Leishmania mexicana, Cysticercus fasciolaris, Trypanosoma cruzi*	
	*Agouti paca*	Wild			Lyssavirus

Carnivora	*Canis familiaris*	Domestic	*Leptospira* spp.^*∗*^, *Ehrlichia canis,* *Rickettsia akari*	*Trypanosoma cruzi,* *Dirofilaria Immitis,* *Dipylidium caninum,* *Ancylostoma caninum, Trichuris vulpis,* *Toxocara canis*	Lyssavirus
	*Felis catus*	Domestic		*Toxoplasma gondii,* *Trypanosoma cruzi*	
	*Spilogale putorius*	Wild			Lyssavirus

Artiodactyla	*Sus domestica*	Domestic	*Leptospira* spp.^*∗*^	*Trypanosoma cruzi * ^*∗*^	
	*Bos taurus*	Domestic	*Leptospira* spp.^*∗*^		
	*Odocoileus virginianus*	Wild			Lyssavirus

Didelphimorphia	*Didelphis virginiana, Didelphis marsupialis, Marmosa mexicana, Philander opossum*	Synantropic/wild	*Leptospira* spp.^*∗*^, *Salmonella enterica, Rickettsia felis*	*Trypanosoma cruzi, Cruzia tentaculata, Turgida turgida*	

Chiroptera	Different species	Wild		*Trypanosoma cruzi*	

^*∗*^Indirect detection (antibodies test).

**Table 2:** Frequency of zoonotic diseases in domestic, synanthropic, and wild animals of the Yucatan Peninsula.

Zoonotic disease informed	Rodents	Dogs	Opossums	Pigs	Cats	Bovids	Bats
	Rate of infection represented in % (number of positives/total sample)						
*L mexicana*	**34.9**						
	**(72/206)**						
	(Van et al., 2009)^2^						

*Leptospira* spp.	**15**	**19**	**5**	**25**		**5.6**	
	**(9/60)**	**(36/192)**	**(4/80)**	**(88/353)**		**(21/375)**	
	(Vado-Solis et al., 2002)^1^	(Vado-Solis et al., 2002)^1^	(Vado-Solis et al., 2002)^1^	(Vado-Solis et al., 2002)^1^		(Vado-Solis et al., 2002)^1^	

*C. fasciolaris*	**7.5**						
	**(31/411)**						
	(Rodriguez-Vivas et al., 2011)^2^						

*T. cruzi*	**8.6**	**15.84**	**11.81**	**5.4**	**34**		**14.67**
	**(16/184)**	**(29/183)**	**(13/110)**	**(15/273)**	**(75/220)**		**(27/184)**
	(López-Cancino et al., 2015)^2^	(Zavala-Velázquez et al., 1996)^1^	(Zavala-Velázquez et al., 1996)^1^	(Jiménez-Coello et al., 2011)^1^	(Jiménez-Coello et al., 2012)^2^		(López-Cancino et al., 2015)^2^
		**7.6%**	**53.9%**				
		**(20/262)**	**(55/102)**				
		(Ucan-Euan et al., 2011)^1^	(Ruiz-Piña and Cruz-Reyes, 2002)^2^				
		**14.76%**					
		**(93/630)**					
		(López-Cespedes et al., 2013)^1^					
		**12.2%**					
		**(45/370)**					
		(Jiménez-Coello et al., 2015)^1^					

*R. felis*	**43.5**		**57.1**				
	**(10/23)**		**(4/7)**				
	(Panti-May et al., 2015)^2^		(Panti-May et al., 2015)^2^				

*D. immitis*		**59.8**					
		**(167/279)**					
		(Caro-González et al., 2011)^2^					

*T. gondii*					**79**		
					**(202/220)**		
					(Castillo-Morales et al., 2012)^2^		

*E. canis*		**8.7**					
		**(27/309)**					
		(Jiménez-Coello et al., 2009)^1^					
		**36%**					
		**(18/50)**					
		(Pat-Nah et al., 2015)^2^					

*A. caninum*		**73.8**					
		**(96/130)**					
		(Rodríguez-Vivas et al., 2011)^2^					
*T. vulpis*		**25.4**(					
		**(33/130)**					
		Rodríguez-Vivas et al., 2011)^2^					

*T. canis*		**6.2**					
		**(8/130)**					
		(Rodríguez-Vivas et al., 2011)^2^					

*D. caninum*		**2.3**					
		**(3/130)**					
		(Rodríguez-Vivas et al., 2011)^2^					

^**1**^Pathogen Indirect detection (Antibodies test). ^**2**^Pathogen direct detection.

**Table 3:** Number of cases of zoonotic diseases officially recorded in humans in the Historical Epidemiological Bulletin (SUAVE) in Yucatan Peninsula that could be associated with human-animal transmission from 2008 to 2019.

Year of report	Zoonotic disease informed						
	Leptospirosis	Toxoplasmosis	Trypanosomiasis	Leishmaniasis	Rocky mountain spotted fever	Other rickettsial diseases	Undefined intestinal diseases
2008	3	NA	NA	NA	NA	NA	192034
2009	5	NA	NA	NA	NA	NA	224259
2010	10	NA	NA	NA	NA	NA	212532
2011	10	NA	NA	NA	NA	NA	243241
2012	7	NA	NA	NA	NA	NA	226718
2013	11	NA	126	NA	NA	NA	233994
2014	12	14	141	148	0	23	219568
2015	17	14	121	208	17	39	232459
2016	6	10	74	201	1	9	204838
2017	3	9	113	416	4	2	339549
2018	5	16	125	307	18	15	323142
2019	15	12	98	675	27	16	320343
Total	104	75	798	1955	67	104	2972677

NA: not applicable.

## Data Availability

The data used to support this study were cited at relevant places within the text as references.
